# PD 1 checkpoint inhibition in solid organ transplants: 2 sides of a coin – case report

**DOI:** 10.1186/s12882-018-1003-5

**Published:** 2018-08-20

**Authors:** Jonathan W. Goldman, Basmah Abdalla, Melody A. Mendenhall, Anthony Sisk, Jaime Hunt, Gabriel M. Danovitch, Erik L. Lum

**Affiliations:** 1 Department of Medicine, Division of Hematology and Oncology, David Geffen School of Medicine rat UCLA, 2020 Santa Monica Blvd, Suite 600, Santa Monica, CA 90404 USA; 2 Department of Medicine, Division of Nephrology, David Geffen School of Medicine at UCLA, Connie Frank Kidney Transplant Center, 200 Medical Plaza, Ste 565, Los Angeles, CA 90095 USA; 3 Department of Pathology, David Geffen School of Medicine at UCLA, 10833 Le Conte Ave, Los Angeles, CA 90095 USA

**Keywords:** Kidney allograft rejection, Nivolumab, Kidney transplant malignancy

## Abstract

**Background:**

The management of malignancy post kidney transplantation includes reduction in immunosuppression and referral to an oncologist management of their malignancy. Recent advances in oncology have resulted in the approval of several classes of drugs with immune-modulatory activity. However, activation of the immune system against malignant cells may precipitate allograft rejection in solid organ transplant recipients.

**Case presentation:**

Herein we present a case of acute kidney allograft rejection in a 50 year old man following administration of the novel immune-modulatory agent nivolumab for the treatment of metastatic squamous cell carcinoma.

**Conclusion:**

The management of malignancy in solid organ transplant recipients requires a heightened awareness of the potential for allograft rejection in this new era of cancer therapeutics.

## Background

The approach to patients who develop malignancy post kidney transplantation has traditionally focused on reduction of overall immunosuppression and the administration of cytotoxic chemotherapy agents under the management of a medical oncologist. However, reduction in immunosuppression to control the malignancy is not without its perils. Reduction in immunosuppression in the kidney transplant recipient with malignancy may result in acute allograft rejection, which may result in reduced renal function and inability to administer appropriate chemotherapy; thereby worsening the patient’s prognosis. This is further compounded by an inability to effectively treat rejection in a patient with active malignancy, as the standard treatment for rejection is a dramatic increase in immunosuppression, which would lead to advanced progression of the malignancy.

More recently, several novel anti-cancer agents targeting the immune check point system have shown improved efficacy compared to standard cytotoxic therapy across multiple tumor types [1–9]. However, these new trials have excluded organ transplant recipients. There is emerging evidence that these agents may precipitate acute allograft rejection in solid organ transplant recipients.

We report the case of a 50 year-old man with end-stage renal disease (ESRD) secondary to polycystic kidney disease (PKD) who underwent a living unrelated donor renal transplant (LURT) followed by immune-mediated graft loss in November 2015 after treatment with nivolumab for metastatic squamous cell carcinoma (SCC). He also achieved a durable anti-cancer benefit from nivolumab, with a partial response that is ongoing for more than 18 months.

## Case report

The patient is a 50 year old male who received a LURT 8 years prior to presentation. He had previously undergone bilateral native nephrectomies 2 months prior to transplant for PKD. His early course was complicated by biopsy-proven acute cellular rejection, vascular type, 5 days after transplant, which was effectively treated with anti-thymocyte globulin and intravenous immunoglobulin. He subsequently went on to enjoy excellent graft function. Initially, he was maintained on standard triple immunosuppression with tacrolimus, mycophenolate mofetil (MMF) and prednisone.

Two years prior to presentation, he developed numerous squamous cell carcinomas of the skin treated with resection and radiation. One of these lesions was an invasive poorly differentiated SCC (Bowen’s type) of the left auricle, requiring auriculectomy and reconstruction. Tumor margins were negative. His immunosuppression was reduced by stopping his MMF.

One year prior to presentation he developed a parotid mass found to be SCC by fine needle aspiration. It was felt that this was a metastatic lesion from the auricular tumor. At this time, he was switched from a dual immunosuppressive regimen of tacrolimus and prednisone to sirolimus (SRL) and prednisone. He underwent a left parotidectomy and neck dissection with pathology showing invasive keratinizing squamous cell carcinoma, poorly differentiated. The tumor was 4.6 cm with lymphovascular and perineural invasion. Surgical margins were negative, but 5 out of 23 periparotid and cervical LNs were positive for metastasis with focal extranodal extension. He underwent radiation therapy and cetuximab. A surveillance PET CT performed 6 months after treatment revealed 5 bilateral pulmonary nodules, which grew over 2 months from 6 mm to 10 mm. He initiated systemic treatment with carboplatin, paclitaxel and cetuximab with minor improvement initially, followed by disease progression in the lungs and mediastinum after 7 months of treatment. He was then treated with gemcitabine, and imaging after 2 months of therapy revealed tumor growth.

A complex discussion was then held regarding symptom-focused palliative care or consideration of novel therapies. Next-generation tumor sequencing was performed on his lung biopsy specimen. Although no clear primary tumor driver was found, 16 genetic abnormalities of possible oncogenic effect were demonstrated, including an EGFR amplification event and a ROS1 mutation of uncertain significance. He enrolled in a clinical trial of the ROS1 inhibitor, entrectinib, but had clinical and radiographic progression within 6 weeks. Other clinical trial options were limited by his history of solid organ transplantation.

With his young age and active lifestyle, the patient opted to proceed with nivolumab 3 mg per kg therapy, understanding the high risk of alloimmune kidney transplant rejection. In preparation, sirolimus was tapered off and prednisone was tapered to 5 mg daily, after which his allograft function remained stable with a creatinine of 1.4 mg/dL. His sirolimus level prior to discontinuation was 6.9 ng/mL.

**Fig. 1:**
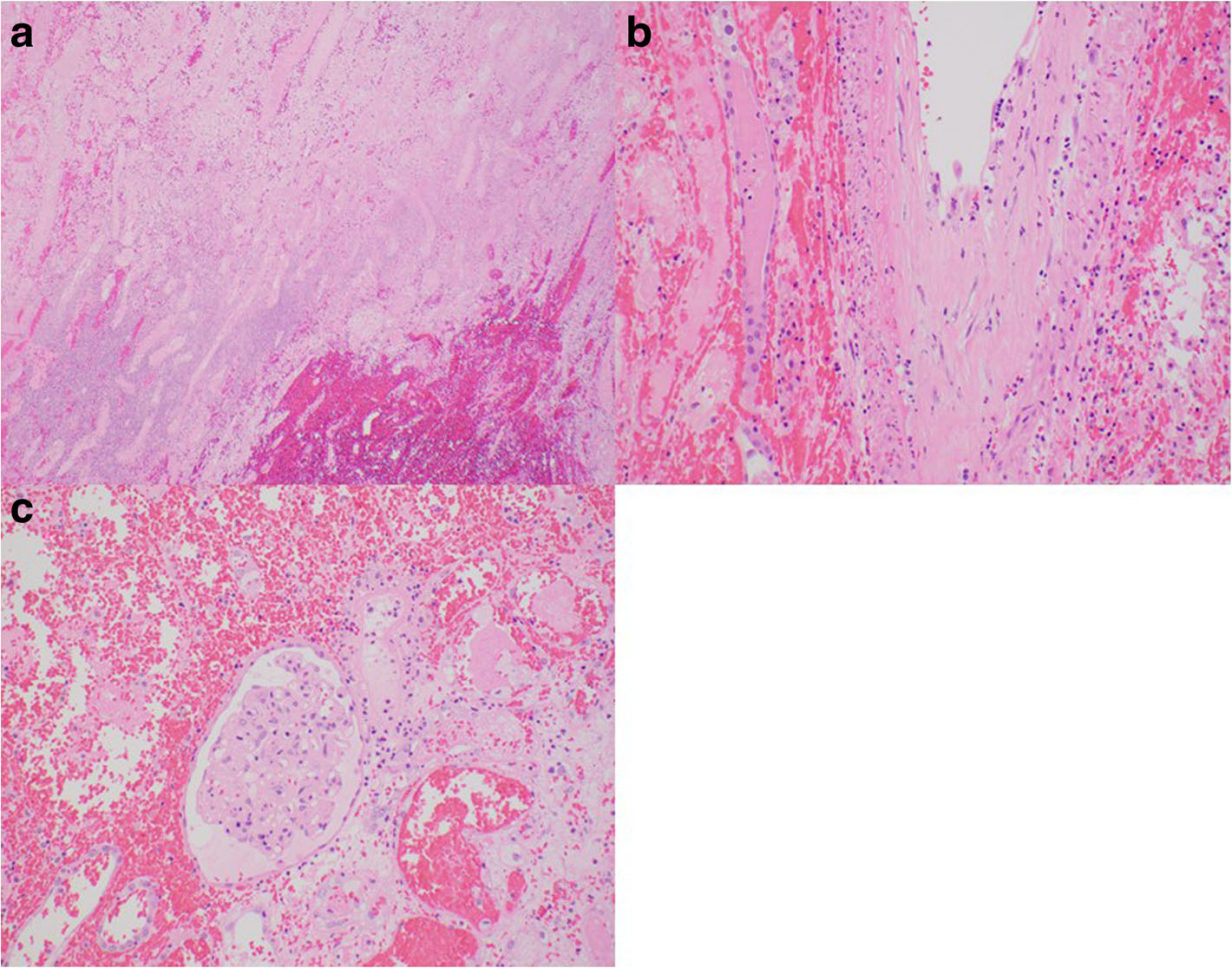
**a** H&E stain. 40× magnification. Infarcted cortex with hemorrhage. From allograft nephrectomy 2 months following rejection episode. **b** H&E stain. 200× magnification. Arcuate artery demonstrating acute endothelialitis and chronic transplant arteriopathy. From allograft nephrectomy 2 months following rejection episode. **c** H&E stain. 200× mag: Partially viable glomerulus with endocapillary hypercellularity (aka: glomerulitis). From allograft nephrectomy 2 months following rejection episode

Thirteen days after receiving the first dose of nivolumab, he presented with low-grade fevers, oliguria and fluid retention. The physical exam demonstrated an enlarged and tender renal allograft and significant lower extremity and peri-orbital edema. Laboratory testing revealed marked acute kidney injury with a creatinine of 4.4 mg/dL. His sirolimus level was noted to be 1 ng/mL and he was treated empirically for acute rejection with a 3 day methylprednisone pulse but without improvement. A renal biopsy was deferred, as he was not a candidate for T-cell depleting therapy with his active malignancy and hemodialysis was initiated for volume overload and electrolyte disturbances. Given the life-threatening nature of his metastatic SCC, the graft was sacrificed and he continued on nivolumab therapy every 2 weeks. Imaging after 4 weeks demonstrated a partial regression in tumor burden and lymphadenopathy. For continued fevers, hematuria and marked allograft pain, an allograft nephrectomy was performed 2 months after stopping his immunosuppression. Histologic evaluation revealed hemorrhagic infarction with features of acute and chronic vascular rejection (Fig. 1).

Now, he continues treatment with nivolumab and most recent imaging 18 months after treatment initiation shows stable tumor regression. He has been maintained on hemodialysis, but has been able to travel and return to an active lifestyle.

## Discussion and conclusions

The susceptibility of transplant recipients to advanced cancers, squamous cell carcinoma in particular, strongly supports the hypothesis that immune dysregulation and deficits in immune surveillance contribute to carcinogenesis. Activation of the immune system to treat malignancy has been known for over a century [10].

More recently, the anti-tumor efficacy of anti-programmed death − 1 (PD 1) and anti-programmed death ligand-1 (PDL1) immune checkpoint inhibition has been demonstrated in patients with metastatic non-small cell lung cancer (NSCLC), melanoma, kidney cancer, Hodgkin lymphoma, head and neck cancer, among other cancers [1–9]. Nivolumab, pembrolizumab, atezolizumab, avelumab and durvalumab have been FDA-approved on the basis of this activity (see Table 1). In settings of immune tolerance, binding of PD1 receptor on T-cells with PDL1 expressed on tumor cells and in the tissue microenvironment results in an inhibitory signal in the effector phase of T-cell response [11] Anti-PD1 and -PDL1 antibodies block this immune checkpoint, thereby leading to not only anti-cancer immune activity but also autoimmune phenomena. Due to non-specific immune activation, this class of drugs has been associated with a 5–10% rate of immune-related toxicity, including pneumonitis, thyroiditis, pancreatitis, hepatitis, and colitis.

**Table 1:** FDA approved PD1 checkpoint inhibitors

Drug Name	Target	Year FDA Approved	Solid Organ rejections
Nivolumab	PD-1	2014	2 cases (NSCLC, Melanoma)
Pembrolizumab	PD-1	2014	2 cases (SCC, Melanoma)
Atezolizumab	PD-L1	2016	None to date
Avelumab	PD-L1	2017	None to date
Durvalumab	PD-L1	2017	None to date

This case unmistakably demonstrates the promise and hazards of checkpoint-inhibitor therapy in solid organ transplant recipients. PD1 blockade provided a significant and durable anti-cancer benefit. Underlying this benefit, the numerous somatic mutations found in his tumor were perhaps indicative of a high mutational burden and may have provided neoantigens that could be recognized by cytotoxic T cells once the PD1-PDL1 inhibitory signal was interrupted. At the same time, the timing and severity of this patient’s rejection leads us to believe it was the direct result of PD-1 inhibitor therapy, although withdrawal of maintenance SRL immunosuppression may have played a role the patient did have detectable low levels at the time of rejection. Four other case reports demonstrate similarly severe cellular rejection (vascular type) with anti-PD1 therapy [12–15]. However, one case report described preserved renal allograft function with preemptive steroids and continued concurrent use of sirolimus with nivolumab, which may provide a potential means to administer anti-PD1 therapy while preserving a transplanted organ [16].

The interplay between anti-cancer immunotherapy and transplant immunosuppression can also be seen with another inhibitory immune checkpoint, the interaction between cytotoxic T-lymphocyte–associated antigen 4 (CTLA-4) and B7. The anti-CTLA-4 antibody, ipilimumab, blocks this interaction and thereby increases T cell activation; it is approved for the treatment of metastatic melanoma. In contrast, belatacept, a fusion protein composed of the Fc receptor of human IgG1 and the extracellular domain of CTLA-4, prevents T cell costimulation and has a demonstrated role in maintenance immunosuppression for kidney transplant recipients. Administration of ipilmumab was recently associated with acute rejection of a kidney allograft [17], and our group has also reported rejection following the administration of the immune modulator lenalidomide in a kidney transplant recipient with multiple myeloma [18].

There is a growing body of literature suggesting that immunotherapy can lead to acute allograft rejection in solid organ recipients. The treating nephrologist and oncologist should be aware of this complication, and patients with cancers amenable to immunotherapy should be counseled extensively on their therapeutic options in order to make the best treatment decision in line with their lifestyle and goals of care. In some cases the benefit of tumor regression may outweigh the unavoidable complication of allograft loss, especially in the setting of advanced metastatic disease. If immunemodulating medications are considered increased allograft monitoring is necessary and a steroid based regimen with continued MTORi therapy may be beneficial.

## Abbreviations

CTLA-4: Cytotoxic T-lymphocyte–associated antigen 4
ESRD: End stage renal disease
LUKT: Living unrelated kidney transplant
MMF: Mycophenolate mofetil
SCC: Squamous cell carcinoma
SRL: Sirolimus
